# miR-146a and miR-155 Expression Levels in Acute Graft-Versus-Host Disease Incidence

**DOI:** 10.3389/fimmu.2016.00056

**Published:** 2016-03-08

**Authors:** Sadaf Atarod, Mohammed Mahid Ahmed, Clare Lendrem, Kim Frances Pearce, Wei Cope, Jean Norden, Xiao-Nong Wang, Matthew Collin, Anne Mary Dickinson

**Affiliations:** ^1^Haematological Sciences, Institute of Cellular Medicine, Newcastle University, Newcastle upon Tyne, UK

**Keywords:** microRNAs, biological markers, acute graft-versus-host disease, allogeneic hematopoietic stem cell transplantation, graft-versus-host disease

## Abstract

Allogeneic hematopoietic stem cell transplantation (allo-HSCT) is a curative treatment for numerous hematological malignancies. However, acute graft-versus-host disease (aGVHD) is still the major complication causing mortality. MicroRNAs (miRNAs) play a significant role in inflammation and have potential as prognostic and diagnostic biomarkers. This study investigated the role of two immune-specific miRNAs (miR-146a and miR-155) as biomarkers for aGVHD incidence in the peripheral blood of allo-HSCT patients prior to disease onset. The study showed that miR-146a and its statistical interaction with miR-155 at day +28 were predictive of aGVHD incidence. Interestingly, the expression levels of miR-146a and miR-155 negatively correlated with the transcription factor, *SPI1* (PU.1gene) mRNA expression.

## Introduction

Graft-versus-host disease (GVHD) is a serious complication of allo-HSCT. Understanding the molecular pathophysiology of GVHD, in particular aGVHD, may aid patient stratification for tailored treatments. MicroRNA (miRNA) investigations have identified biomarker signatures for numerous diseases such as cancer and rheumatoid arthritis and more recently for GVHD (1–4). Since miR-146a and miR-155 are both involved in the adaptive and the innate immune system (5, 6), we assessed their role in allo-HSCT. miR-146a is essential for Treg function (7). TNF receptor-associated factor 6 (TRAF6) and interleukin-1 receptor-associated kinase 1 (IRAK1) are miR-146a targets, and their expressions are regulated in a negative feedback-loop via the TLR-4 (Toll-like receptor) signaling pathway and NFκB activation (8, 9). IFN regulatory factor 5 (IRF5) and signal transducer and activator of transcription 1 (STAT1) are also known targets of miR-146a (10). Tang et al. (10) showed that overexpression of miR-146a lowered STAT1 and IRF-5 at both the molecular and the protein level. Moreover, it has been shown in mice that miR-146a depleted Treg cells have high levels of Stat1 and this results in IFN-γ mediated autoimmunity (7). In a recent GVHD murine study, miR-146a was under-expressed in the aGVHD group and the expression correlated with disease severity, while its target, Traf6, was overexpressed, suggesting the involvement of NFκB activity (4).

miR-155 is required for normal B and T lymphocyte function in humans (11). miR-155 is necessary for CD8+ T cell proliferation, and its deficiency can lead to the overexpression of STAT1, leading to down regulation of proliferation of T cells (12, 13). In a GVHD patient cohort (*n* = 5), miR-155 was overexpressed in the gut of aGVHD patients while its expression was absent in normal gut (1). Recently, a murine study has shown that miR-155 expression activates inflammasomes via the migration of dendritic cells (14). Thus, inhibiting miR-155 expression may reduce GVHD severity.

In addition to TRAF6, IRAK1, STAT1, and IRF5 the important transcription factor, SPI1 (PU.1), which functions in hematopoietic differentiation (15), also regulates the expression of miR-146a and miR-155 (16). In a murine study, it was demonstrated that Spi1 regulates the expression of both miR-146a and miR-155 by binding to their pre-miRNA loci (17). Hence, the primary focus of this investigation was miR-146a and miR-155 expression levels because of their known functions in both the adaptive and innate immune system. The aims of this investigation were: (1) to assess whether miR-146a and miR-155 were associated with aGVHD incidence and severity as well as with clinical outcomes including overall survival (OS) and relapse, (2) to establish a statistical model for predicting aGVHD incidence and severity using the miRNA expression levels and (3) to examine the correlation between miR-146a and miR-155 expression with their targets (IRAK1, TRAF6, STAT1-α, IRF5, and *SPI1*).

In summary, this research has shown that miR-146a and miR-155 are lower in patients who go on to develop aGVHD (grade I–III). *SPI1* is regulated by both miRNAs during the early stages of inflammation, and there was no correlation between the miRNAs and their targets.

## Materials and Methods

### Ethics and Consent

The project was approved by the Newcastle and North Tyneside I Research Ethics Committee (Stemdiagnostics: 07/H0906/131). All the investigations were conducted in accordance with the Helsinki Declaration. The patients gave their informed written consent for whole blood, serum sample collection, testing, and analyses.

### Patient Study Cohort

The cohort consisted of 54 patients who had undergone allo-HSCT (2010–2013) with either Human Leukocyte Antigen (HLA) matched siblings or matched unrelated donors (Table 1). There were only seven cases of “female donor-to-male recipient” and overall there were 21 patient deaths (events) and 33 patients who were alive at the end of the study (censored cases). This study included only patients who developed no aGvHD or who developed aGVHD within the first 100-days post-transplantation (classic aGVHD). Patients who received donor lymphocyte transfusions, or started immunosuppression withdrawal, before 100 days were excluded. None of the patients had developed GVHD prior to day 28. Classic maximum aGVHD grade [disease onset: median = 62 days (range: 33–100)] was assessed as per the NIH consensus (18) [grade 0: without (*n* = 26), grade I: mild (*n* = 17), grade II: moderate (*n* = 10) and grade III: severe aGVHD (*n* = 1)]. The incidence of chronic GVHD (cGVHD) was significantly associated with prior aGVHD (Table 1). In the patient group who did not develop aGVHD (grade 0), 50% had died from disease relapse and 30% due to infections, while in patients with aGVHD grades I–III, 37% had died of relapse and 46% due to infections. In total two patients had died of cGVHD and an additional two due to other complications. Whole blood was collected from patients 7 days before HSCT (*n* = 35), at 28 days (*n* = 54) and at 3 months (*n* = 34) post-transplantation. At day +28, none of the patients were on steroids nor had they received donor lymphocyte infusion, and all patients survived until day 100. All patients had received alemtuzumab.

**Table 1:** **Frequency of patient characteristics in patients with and without incidence of aGVHD grades I–III**.

Cohort characteristics (*n* **=** 54)		No aGVHD (grade 0) (%) (*n* **=** 26)		Yes aGVHD (grade I–III) (%) (*n* **=** 28)		Difference (*p*-value)

		No.	%	No.	%	
Patient age (median years)		54.53 (19.85–67.63)				0.741
cGVHD	No	11	73	4	27	**0.011**
	Yes	8	31	18	69	
Patient gender	Female	12	63	7	37	**0.155**
	Male	14	40	21	60	
Donor gender	Female	8	57	6	43	0.533
	Male	16	44	20	56	
Graft source	BM	3	50	3	50	1
	PBSC	23	48	25	52	
Underlying disease	ALL	3	43	4	57	**0.128**
	AML	4	27	11	73	
	CLL	2	100	0	0	
	CML	1	100	0	0	
	MDS	9	64	5	36	
	MM	2	67	1	33	
	MPS	2	100	0	0	
	NHL	3	33	6	67	
	SAL	0	0	1	100	
Regimen	Myeloablative	3	27	8	73	**0.179**
	RIC	23	53	20	47	
Protocol	Flu Mel	22	55	18	45	**0.133**
	Flu Bus	3	60	2	40	
	Cy	1	17	5	83	
	FluCycloAlem	0	0	3	100	
CsA prophylaxis at day +28 (ng/ml)		159 (68–333)				0.795
Alemtuzumab	30 mg	13	52	12	48	0.497
	60 mg	12	43	16	57	
	90 mg	1	100	0	0	
Relationship	SIB	11	55	9	45	0.574
	MUD	15	44	19	56	
Patient CMV status	Negative	11	46	13	54	0.791
	Positive	15	50	15	50	
Donor CMV status	Negative	17	53	15	47	0.418
	Positive	9	41	13	59	
HLA Class I mistmatches	None	24	47	27	53	0.604
	One	2	67	1	33	
HLA Class II mismatches	None	17	61	11	39	**0.096**
	One	7	41	10	59	
	Two	2	22	7	78	
Survival status	Alive	16	48	17	52	1
	Dead	10	48	11	52	
Relapse status	No	18	47	20	53	1
	Yes	8	50	8	50	
Disease status at transplant	CR	2	100	0	0	0.437
	CR1	11	52	10	48	
	CR2	4	33	8	67	
	CR3	2	40	3	60	
	Unt	4	67	2	33	

*Fisher’s exact test was used to estimate the difference in frequencies between the two different aGVHD groups. Significant *p*-values are in bold and underlined (*p* ≤ 0.2). cGVHD, chronic GVHD; BM, bone marrow; PBSC, peripheral blood stem cells; ALL, acute lymphocytic leukemia; AML, acute myeloid leukemia; CLL, chronic lymphocytic leukemia; CML, chronic myeloid leukemia; HD, Hodgkin’s disease; MDS, myelodysplastic syndrome; MM, multiple myeloma; MPS, myeloproliferative syndrome; NHL, Non-Hodgkin’s lymphoma; SAL, secondary acute leukemia; RIC, reduced intensity conditioning; Flu, fludarabine; Mel, melphalan; Bus, busulfan; Cy, cytrabine; Cyclo, cyclophosphamide; TBI, total body irradiation; CsA, cyclosporin; CMV, cytomegalovirus; CR (1–3), complete remission; Unt, untreated; SIB, sibling; MUD: matched unrelated donor*.

### Total RNA Extraction from Whole Blood

Peripheral blood was collected in PAXgene Blood RNA Tubes (19). The samples were stored at −20°C prior to extraction. Total RNA was extracted using the PAXgene Blood miRNA kit (Qiagen/BD Company) as per the manufacturer’s guidelines. Total RNA quality was determined using the Nanodrop 1000 (Agilent, USA).

### Reverse Transcription and Real-Time PCRs for MicroRNAs and for Gene Expression

For miRNA expression assays (Life Technologies, USA): 1–10 ng of total RNA was reverse transcribed (RT) in a 15 μl reaction using Taqman specific reverse transcription primers [hsa- miR-146a-5p (Assay ID: 000468), miR-155-5p (Assay ID: 000479), and SNORD48 (Assay ID: 001006)] and the Taqman miRNA Reverse Transcription kit. The RT PCR step was performed as per the manufacturer’s guidelines. miRNA-specific cDNAs were used to run qPCR using miRNA Taqman primer-probe sets and TaqMan^®^ Gene Expression Master Mix. SNORD48 was used for normalization.

For gene expression assays, total denatured RNA (5 min at 65°C) was RT using equal volume of master mix (1:1) consisting of: random hexamers (Thermo Scientific, USA), dichloro-diphenyl-trichloroethane (Invitrogen, USA), first-strand buffer (Invitrogen), dNTPs (Roche Diagnostics, USA), reverse transcriptase (Invitrogen), and Rnase inhibitor (Promega, USA). The reaction mix was incubated at 37°C for 2 h and 10 min at 65°C. cDNA was used to run qPCR for the miRNA targets [*TRAF6* (Hs00371512_g1), *IRAK1* (Hs01018347_m1), *STAT1-*α** (Hs01014003_m1), *IRF5* (Hs00158114_m1), and *SPI1* (Hs02786711_m1)]. GAPDH (Assay ID: 4352934E) was used as the reference gene. All the qPCRs were run in triplicate along with no-template controls on the 7900HT Fast Real-Time PCR System (Life Technologies), using standard thermal cycling conditions. The comparative delta-delta C_q_ (cycle quantification) method was used to calculate relative fold-changes, and all data were log_2_ transformed.

### MicroRNA Protein Target Detection

Peripheral blood was collected into vacutainer tubes coated with potassium Ethylenediaminetetraacetic acid (EDTA) (Becton Dickinson). The blood clot was allowed to retract for 4–7 h at 4°C and then centrifuged at 500 g. Sera were then collected and stored at −80°C till use. Commercially available Enzyme-Linked Immunosorbent Assay (ELISA) kits were used for the detection of miRNA protein targets [TRAF6 (CSB-E14078h), IRAK1 (CSB-E09933h), STAT1-α (CSB-E11755h), and IRF5 (CSB-EL011820HU)]. All the steps were performed as per the manufacturer’s guidelines (CUSABIO, China).

### Statistical Analyses

Analyses were performed per time-point (day −7, day +28, and 3 months). Statistical significance level was set at *p* ≤ 0.05 and for trend tests at *p* ≤ 0.1.

#### Acute GVHD Incidence

Primary univariate analysis of the cohort using Fisher’s exact test was performed to identify clinical risk factors associated with aGVHD incidence. Statistical significance for initial inclusion of candidate risk factors was set at *p* ≤ 0.2, as per the statistical guidelines from the European Society for Blood and Marrow Transplantation (20).

To obtain the best model fit, and thus the most accurate predictions, aGVHD incidence, which is recorded using a binary scale (0 = no aGVHD or 1 = aGVHD grades I–III), was assessed using a forward step-wise, multiple binary logistic regression procedure (SPSS version 21.0). This uses the logit function to model the probability (or “odds”) of a patient having or not having aGVHD, given their clinical background and miRNA results. In this procedure, the significant clinical risk factors were considered as covariates, as well as the effects of miR-146a and miR-155 expression levels, and to give the best model fit, their statistical interaction was also considered (21, 22).

#### Survival Analyses

Univariate Kaplan-Meir and Cox regression were used to determine candidate clinical risk factors for time-dependent outcomes OS and relapse (*p* ≤ 0.2). Multivariate Forward-LR Cox regression analysis was performed to model these outcomes (SPSS version 21.0).

#### Correlation of miRNAs with Targets

Spearman correlation was performed to test for strength of association between miRNA expression levels and their targets. The correlation was performed separately for those who never developed aGVHD (grade 0) and those who went on to develop grade I–III aGVHD.

Kruskal–Wallis analysis of variance was performed when there were three or more groups in the comparison, and Dunn’s *post hoc* test was used to safeguard against false positives (GraphPad Prism version 5).

## Results

### The Statistical Interaction between miR-146a and miR-155 was Significantly Associated with aGVHD Incidence at Day +28 Post-allo-HSCT

**Figure 1:**
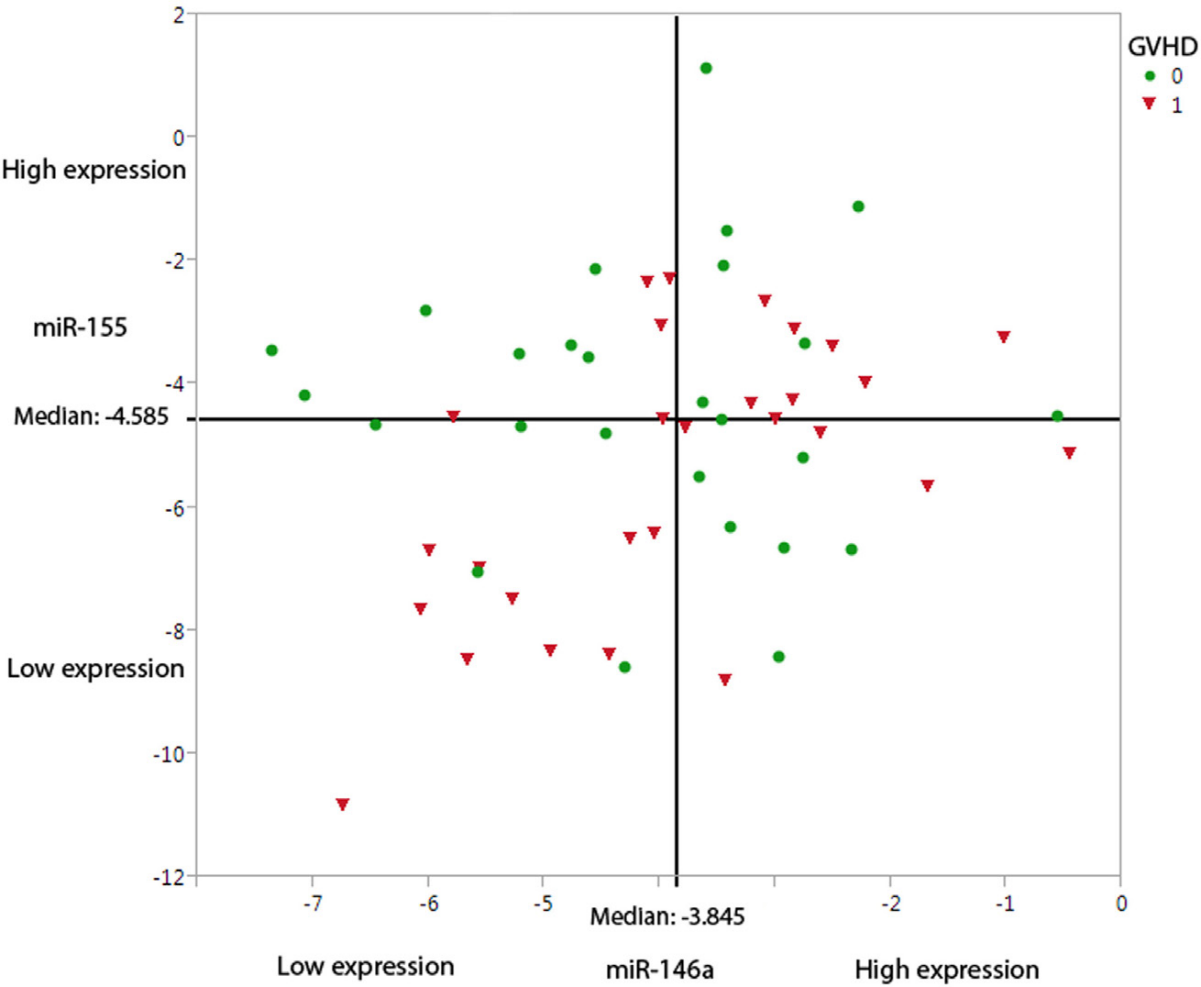
**Under-expression of miR-146a-5p and miR-155-5p leads to a higher probability of aGVHD incidence**. The graph demonstrates the miR-146a-5p and miR-155-5p expression levels at day +28, dichotomized based on aGVHD incidence (0 = no GVHD and 1 = aGVHD grades I–III). The straight-line shows the median expression levels in this cohort (*n* = 54) for miR-146a-5p (−3.845) and miR-155-5p (−4.585).

A hierarchical model was built using a forward stepwise procedure including significant clinical risk factors, miR-146a and miR-155 expression levels, and their interaction, separately for each time-point. Only day +28 data produced a significant model fit. The clinical risk factors initially determined to be of possible relevance (*p* ≤ 0.2) in predicting aGVHD incidence, and which were included in the step-wise procedure were: patient gender, underlying disease, HLA class II mismatches, conditioning regimen, and transplant protocol (Table 1). Results showed that the clinical factors were not significant once both miRNA expression levels at day +28 were included in the model. The final model that resulted in the best overall fit and predictive ability included a significant statistical interaction between the two miRNAs (Table 2: the “main effect” of each miRNA was included to enforce model hierarchy; only the main effect of miR-146a was statistically significant). By simple graphical or tabular examination of the raw data, we can see that this statistical interaction represents a biological synergy: if both miR-146a and miR-155 were expressed at low levels, this was associated with a higher incidence of aGVHD (Figure 1; Table 3). The classification matrix illustrating the predictive ability of the model showed that 69% of the patients were accurately classified as without aGVHD (grade 0) and 75% as with aGVHD incidence (grades I–III).

**Table 2:** **Statistical hierarchical model fitting for miR-146a-5p and miR-155-5p at day +28 postallo-HSCT**.

MicroRNAs	Sig.	Odds ratio (95% CI)
miR-155-5p	0.069	0.34 (0.10–1.09)
miR-146a-5p	0.016	0.15 (0.03–0.70)
miR-155-5p*miR-146a-5p	0.025	0.71 (0.53–0.96)

*A step-wise multivariate binary logistic generalized linear model was used for the prediction of aGVHD incidence*.

**Table 3:** **Summary of miRNA expression at day +28 dichotomized into high (↑ – above median level of expression) and low (↓ – below median) levels with regards to grade 0 and grades I–III aGVHD showing that when both miRNAs are under-expressed, there is a higher probability of having aGVHD (highlighted)**.

MicroRNA expression levels		GVHD grades (Number of patients)	
miR-146a-5p	miR-155-5p	Grade 0	Grade I–III
↑	↑	8	7
↓	↓	5	10
↓	↑	7	5
↑	↓	6	6

As the clinical risk factors were not significant in the final model, the equation that can be used for predicting the probability of a patient having aGVHD as a function of their miR expression levels is:

$$\begin{array}{l} \text{probability (patient has aGVHD},\text{ given miRNA levels)}\\ =\frac{\exp(\beta_{0}+\beta_{\text{1}}\text{Log}146\text{a}+\beta_{2}\text{Log}155+\beta_{3}(\text{Log}146\text{a}\times \text{Log}155)}{1-[\exp(\beta_{0}+\beta_{1}\text{Log146a}+\beta_{\text{2}}\text{Log155}+\beta_{\text{3}}\text{(Log146a}\times \text{Log}155)]} \end{array}$$

β_0_ = intercept coefficient and β_1–3_ = regression coefficients, Log146a = −log2(miR-146a expression), Log155 = −log2(miR-155 expression).

In this study the probability (patient has aGVHD, given miRNA levels)

$$=\frac{0.0022}{\exp[1.91\text{ Log146a}+\text{1.091 Log155}+\text{0.342(Log146a}\times \text{Log}155)]}$$

Log146a = log_2_ (miR-146a expression), Log155 = log_2_(miR-155 expression).

In contrast, aGVHD severity (grades II–III) was not predictable from miRNA expression levels for this cohort, possibly because the majority of the patients had either none or grade I aGVHD (79.6%) post day +28.

### miR-146a and miR-155 Expression in Whole Blood was Not Associated with Relapse

Patient gender, transplant protocol, donor cytomegalovirus status, aGVHD incidence, and mismatches in HLA Class I were determined as significant clinical risk factors for inclusion in the model building. The multivariate Cox regression model showed that only mismatches in HLA class I was marginally predictive of patient risk of relapse [*p* = 0.048, HR = 4.744 (95% CI: 1.016−22.154)], and that the expression levels of both miR-146a and miR-155 were not significantly associated with relapse.

### A Positive Correlation Was Observed between the miR-146a Targets at the mRNA Level

The step-wise logistic regression results, discussed previously, indicated that miR-146a was best for predicting aGVHD incidence; thus, to further understand its role in GVHD, its established targets (*TRAF6*, *IRAK1*, *STAT1-*α** and *IRF-5*) were quantified at the mRNA and protein levels. In patients without aGVHD [grade 0 (*n* = 26)], there was a trend toward a negative correlation between miR-146a and *TRAF6* mRNA expression (*r*_s_ = −0.37, *p* = 0.061) only at day +28 post-HSCT. Since all the miRNA targets are involved in the same pathways, we assessed whether their levels correlated with each other at both pre- and post allo-HSCT time-points. A statistically significant (*p* ≤ 0.002) positive correlation was observed between all the targets in the entire patient cohort (Table 4).

**Table 4:** **Spearman correlation showed significant positive correlation between all the mRNA targets in whole blood collected from allo-HSCT patients (*n* = 51–54) on day +28**.

Spearman correlation		*TRAF6*	*IRAK1*	*STAT1-*α	*IRF5*	*SPI1*	miR-146a
*IRAK1*	*r*_s_	0.800					
	*p*-value	**0.000**					
*STAT1-*α	*r*_s_	0.770	0.580				
	*p*-value	**0.000**	**0.000**				
*IRF5*	*r*_s_	0.680	0.700	0.650			
	*p*-value	0.000	0.000	0.000			
*SPI1*	*r*_s_	0.480	0.320	0.540	0.390		
	*p*-value	**0.000**	**0.024**	**0.000**	**0.005**		
miR-146a	*r*_s_	−0.160	−0.120	0.000	−0.090	−0.450	
	*p*-value	0.264	0.392	0.981	0.542	**0.016**	
miR-155	*r*_s_	−0.170	−0.110	−0.120	−0.100	−0.530	0.220
	*p*-value	0.229	0.438	0.394	0.504	**0.004**	0.114

*Significant negative correlation was present for both miR-146a-5p and miR-155-5p with *SPI1*. *r*_s_, Spearman correlation coefficient. Significant p-values are in bold and underlined (p *≤* 0.05)*.

In addition, correlation between miR-146a and its targets was also assessed in the aGVHD patient cohort (grades I–III). Measurements at day +28 showed that there was no relationship between miR-146a and its targets, but there was statistically significant (*p* ≤ 0.02) positive correlation observed between all the targets (data not shown). At 3 months post-transplantation, there was a significant negative correlation (*r*_s_ = −0.69, *p* = 0.019) between miR-146a and *IRF5* mRNA expression. At the same time-point, *TRAF6* and *IRAK1* mRNA expression positively correlated with each other (*r*_s_ = 0.86, *p* = 0.001).

### IRF5 mRNA Expression Positively Correlated with Protein Levels Measured in Whole Blood at Day +28 in aGVHD Patients

The target protein levels were quantified in a representative subset of the cohort (grade 0, *n* = 5; grades I–III, *n* = 10). Only STAT1-α and IRF5 proteins were detectable by ELISA. It may be that the TRAF6 and IRAK1 protein levels were below the sensitivity of the assay [detection range (picogram per milliliter), for TRAF6: 39-2500 and IRAK1: 312-2000].

The pre-transplant STAT1-α and IRF5 protein levels neither correlated with their mRNA expression nor with miR-146a-5p expression levels (*p* > 0.05) (data not shown). The day +28 protein levels of STAT1-α and IRF5 in the aGVHD grade 0 cohort significantly positively correlated with each other (*r*_s_ = 0.90, *p* = 0.037). At 3 months post-transplantation, there was no correlation between miR-146a and its protein targets. In the aGVHD cohort (grades I–III), at Day +28, there was a trend for positive correlation between miR-146a-5p and STAT1-α protein levels (*r*_s_ = 0.53, *p* = 0.092). A similar correlation was observed between *STAT1-*α mRNA and its serum protein level (*r*_s_ = 0.57, *p* = 0.068). There was also a significant positive correlation (*r*_s_ = 0.71, *p* = 0.009) between IRF5 mRNA and its serum protein level. At 3 months post-transplantation, there was a significant negative correlation (*r*_s_ = −0.69, *p* = 0.019) between miR-146a-5p and IRF5. Some patients at 3 months were receiving steroids so this result must be interpreted with caution.

### miR-155 and miR-146a Expression Correlated with the Transcription Factor, SPI1

**Figure 2:**
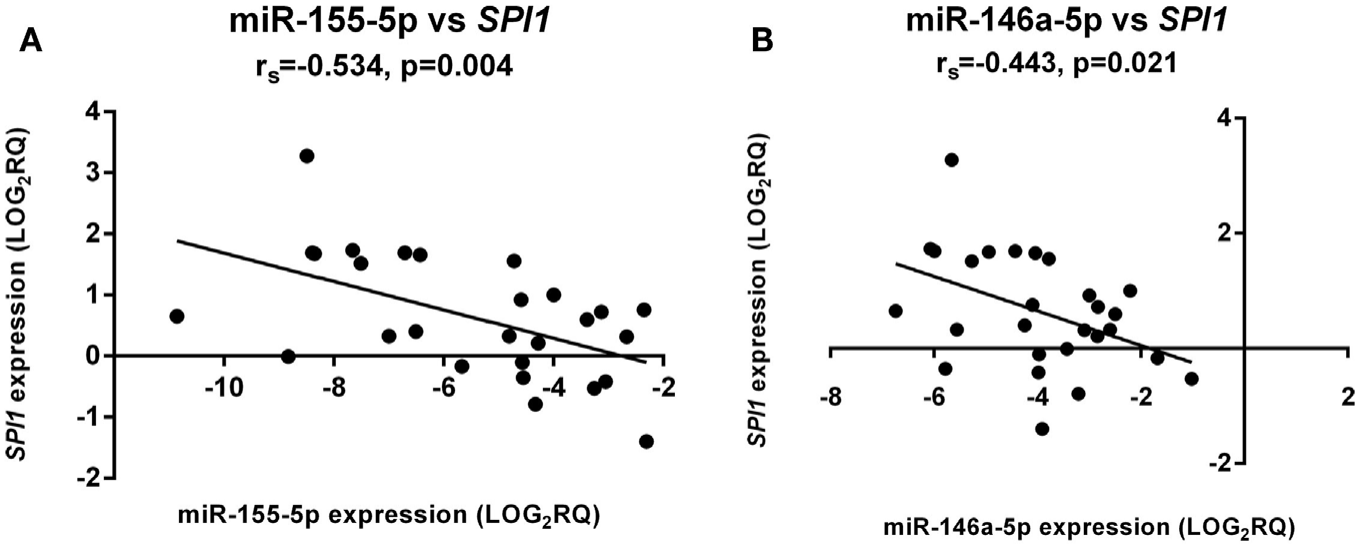
**miR-155-5p and miR-146a-5p expression directly correlated with SPI1 mRNA levels**. There was a significant negative correlation for both **(A)** miR-155-5p and **(B)** miR-146a-5p expression. The correlation was only significant in aGVHD patients with grades I–III (*n* = 27). The *x*-axis shows the miRNA expression levels and the *y*-axis the *SPI1* mRNA levels. Spearman correlation was used to test for correlation. Spearman rho: *r*_s_. Significance level was set at *p* ≤ 0.05.

In the aGVHD patient cohort (grades I–III) only, miR-155-5p expression was significantly negatively correlated with its target *SPI1* in whole blood samples at day +28 (Figure 2A). In the same cohort, there was a significant negative correlation between miR-146a-5p and *SPI1* (Figure 2B).

## Discussion

miR-146a and miR-155 have been shown to be indispensable in regulating immune-related pathways (6, 7, 23, 24). It is known in HSCT that miR-155-5p is overexpressed when target organ damage has already occurred in the patient (1). It is also known that graft-versus-tumor effect is required to prevent disease relapse (25). Thus, tailoring treatments to maintain a balance between GVHD and graft-versus-tumor effect are important post-HSCT.

This study aimed at assessing whether the expression of the two miRNAs could be used to predict aGVHD prior to disease onset. This knowledge could make clinical interventions possible prior to disease onset and, therefore, prevent the complications associated with aGVHD. Understanding the miRNA regulation involved in the pathophysiology of aGVHD could also be reflective of the GVH reaction and in particular the essential graft-versus-tumor effect, directing new therapies for improved HSCT disease-outcome.

The data models used in this study looked at both the main effects and the statistical interaction between miR-146a and miR-155 in predicting aGVHD incidence. In many biological investigations, in particular in the field of allo-HSCT, statistical interactions are often overlooked even though they are one of the keys to better fitting models (and thus better predictions) and to the better understanding of biological relationships. This investigation is one of the few studies where this method has been utilized for aGVHD incidence prediction.

The statistical interaction of miR-146a and miR-155 expression levels at day +28 post-transplant was significant in predicting aGVHD incidence, and investigation of the data revealed the conditional regulation mechanism wherein when both miRNAs were expressed at lower levels, there was a higher incidence of aGVHD. The statistical interaction observed is in support of the differential “check-point” mechanism shown by Schulte et al. (26). Using various immune system stimulators such as lipopolysaccharides that trigger the expression of miR-146a (8), the authors showed that miR-146a was upregulated first, thus initiating the inflammatory response. miR-155 expression was only altered when miR-146a levels of inflammatory tolerance had been surpassed (26). This may explain why higher miR-155 expression levels have been observed in both the serum and gut of patients at the time of aGVHD onset, which is the effector phase of the GVHD cycle (1, 27, 28). Our results support the very recent aGVHD study in the mouse that showed miR-146a expression levels were lower in severe aGVHD cases (4). This may suggest that overexpression of miR-146a prevents an inflammatory response and may have a protective role in innate immunity (23).

miR-146a expression affects the expression of TRAF6 and IRAK1 at the protein level rather than at the mRNA-level (29). But in this study using ELISAs, the protein level of IRAK1 and TRAF6 were undetectable in the sera of allo-HSCT patients. IRF5 and STAT1-α proteins were detected in the allo-HSCT patients, but they did not correlate with miR-146a expression levels. *IRF5* mRNA expression positively correlated with its protein levels at day +28 in patients who went on to develop aGVHD but not in patients who did not develop aGVHD. Not surprisingly, a positive correlation was present among miR-146a-5p targets at the mRNA level at both pre- and post-allo-HSCT time-points. It is at the augmented inflammatory stage that the miRNA expression reaches a level that impacts the expression levels of their targets. This may be the reason why no correlation was seen between miR-146a-5p and its targets pre-aGVHD onset, while the same miRNA has been shown to regulate Traf6 expression at aGVHD onset (4).

Mice deficient in miR-146a develop autoimmune disorders and die prematurely (29). Low miR-146a expression could result in an inflammatory phenotype and impact survival (30). This may suggest that patients with lower miR-146a-5p expression would have more graft-versus tumor effect due to exacerbated GVH reaction, but this could lead to fatal GVHD. When the statistically significant clinical risk factors were included in the study, results showed that only HLA class I mismatches were associated with risk of death (data not shown). This finding may suggest that miR-146a expression was not associated with OS when clinical risk factors were included in the analysis. However, there were only three patient-donor pairs with HLA class I mismatch and thus this finding is preliminary and must be considered with caution.

The master transcription factor *SPI1* (31) controls miR-155 expression (17). Both miRNAs are predicted to target *SPI1* according to microrna.org (32). *SPI1* controls the expression of both miRNAs in HSCs (17). Spi1 binds to pre-miR-146a locus permanently to regulate its expression and to miR-155 locus only during hematopoietic stem cell differentiation (17). Our results may suggest that during GVH reaction both miRNAs are triggered by chemokines and cytokines, which lead to their regulation of *SPI1* rather than their activation by it (17, 33).

**Figure 3:**
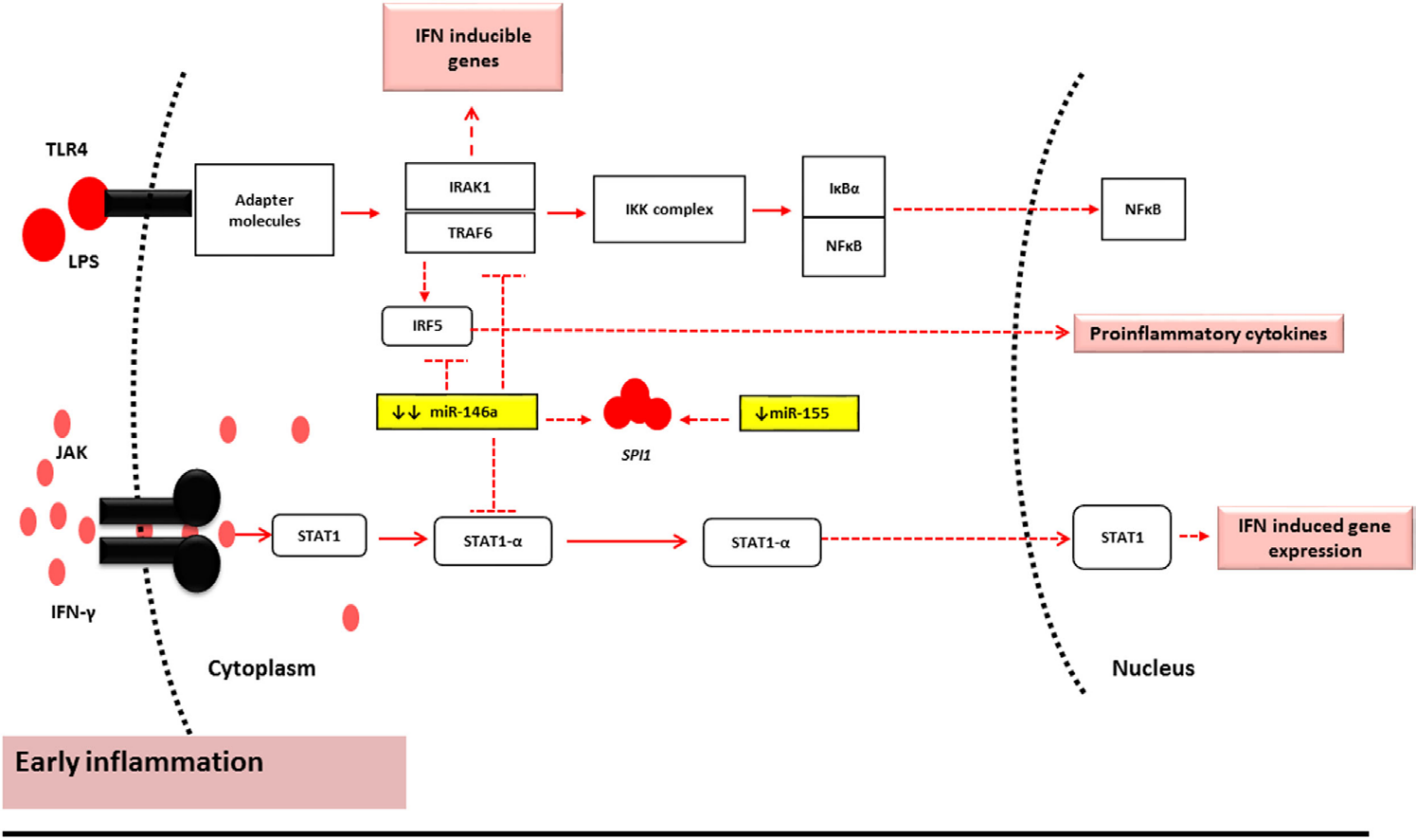
**miR-146a-5p and miR-155-5p mechanism of action under normal conditions and early stages of inflammation** (2, 9, 34). During the early stages of inflammation in GVHD, the TLR and Janus Kinase (JAK)-STAT1 pathway are activated by LPS and IFN-γ, respectively. Under such conditions, miR-146a-5p and miR-155-5p have reduced expression levels. This results in minor alterations of the mRNA target levels and may therefore represent subclinical early inflammatory GVHD.

The findings of this study may suggest that miR-146a-5p and miR-155-5p act as “paramedics” in the initial phases of inflammation due to their characteristic sensitivity. Thus, their levels are deregulated pre-aGVHD onset or sub-clinically. However, once the limit of miR-146a-5p tolerance is breached, and miR-155 is overexpressed to compensate, the immune system is alarmed (26). It is at this heightened inflammatory stage that their expression reaches a level that impacts the expression levels of their targets. This may be the reason why no correlation was seen between miR-146a-5p and its targets pre-aGVHD onset while the same miRNA has been shown to regulate TRAF6 expression at the time of aGVHD onset (4). This also explains the significant main effect of miR-146a-5p and its interaction with miR-155-5p observed in this study pre-aGVHD disease onset (Figure 3).

Since there is subclinical manifestation of aGVHD, the ­expression levels of both miRNAs could be early markers of aGVHD incidence.

The results need to be validated in a larger multi-center cohort and in patients with more severe aGVHD grades III–IV to ascertain whether both miRNAs could be used as predictive biomarkers for severity.

## Author Contributions

SA designed and performed the research, data analyses and wrote the manuscript. MA: performed parts of the research. JN, X-NW, and AD: designed the study and commented on manuscript. CL and KP: contributed to statistical analyses and data interpretation. CL: collated the clinical information. MC: data interpretation, contributed clinical information and samples for the study.

## Conflict of Interest Statement

The authors declare that the research was conducted in the absence of any commercial or financial relationships that could be construed as a potential conflict of interest.
